# Role of the small GTPase Rab27a during Herpes simplex virus infection of oligodendrocytic cells

**DOI:** 10.1186/1471-2180-12-265

**Published:** 2012-11-19

**Authors:** Raquel Bello-Morales, Antonio Jesús Crespillo, Alberto Fraile-Ramos, Enrique Tabarés, Antonio Alcina, José Antonio López-Guerrero

**Affiliations:** 1 Centro de Biología Molecular Severo Ochoa, CSIC-UAM, Nicolás Cabrera 5, Cantoblanco, 28049, Madrid, Spain; 2 Universidad Complutense de Madrid, Facultad de Medicina, Ciudad Universitaria, 28040, Madrid, Spain; 3 Universidad Autónoma de Madrid, Facultad de Medicina, Arzobispo Morcillo 4, 28029, Madrid, Spain; 4 Instituto de Parasitología y Biomedicina López Neyra, CSIC, Parque Tecnológico de Ciencias de la Salud, Avenida del Conocimiento s/n, 18100, Armilla, Granada, Spain

**Keywords:** HSV-1, Oligodendrocytes, Rab27a, Viral egress, Morphogenesis, Tegument

## Abstract

**Background:**

The morphogenesis of herpes simplex virus type 1 (HSV-1) comprises several events, of which some are not completely understood. It has been shown that HSV-1 glycoproteins accumulate in the *trans*-Golgi network (TGN) and in TGN-derived vesicles. It is also accepted that HSV-1 acquires its final morphology through a secondary envelopment by budding into TGN-derived vesicles coated with viral glycoproteins and tegument proteins. Nevertheless, several aspects of this process remain elusive. The small GTPase Rab27a has been implicated in regulated exocytosis, and it seems to play a key role in certain membrane trafficking events. Rab27a also seems to be required for human cytomegalovirus assembly. However, despite the involvement of various Rab GTPases in HSV-1 envelopment, there is, to date, no data reported on the role of Rab27a in HSV-1 infection.

**Results:**

Herein, we show that Rab27a colocalized with GHSV-UL46, a tegument-tagged green fluorescent protein-HSV-1, in the TGN. In fact, this small GTPase colocalized with viral glycoproteins gH and gD in that compartment. Functional analysis through Rab27a depletion showed a significant decrease in the number of infected cells and viral production in Rab27a-silenced cells.

**Conclusions:**

Altogether, our results indicate that Rab27a plays an important role in HSV-1 infection of oligodendrocytic cells.

## Background

Several evidences indicate that a viral infection could be involved in the aetiology of demyelinating diseases, such as Multiple Sclerosis (MS) [1]. Several members of the *Herpesviridae* family, including Herpes simplex virus type 1 (HSV-1), have been suggested as possible causes of this pathology [2,3]. Oligodendrocytes, the myelin-producing glial cells in the central nervous system, have proven to be susceptible to this alphaherpesvirus *in vivo*[4-7] and in cultured cells [8]. Therefore, to deepen the knowledge on HSV-1 infection of myelinating cells, will contribute in clarifying relevant aspects of demyelination aetiology.

HSV-1 is a highly prevalent neurotropic human pathogen that can infect and establish latency in neurons. HSV-1 can cause, in certain circumstances, severe pathologies such as keratoconjunctivitis and encephalitis. Following primary infection of epithelial cells, virions spread to neurons and establish latent infections in the trigeminal ganglia. The morphogenesis of HSV-1 has been broadly studied [9-11], but several events of this complex process remain unsolved. Viral transcription, replication, packaging of the new viral particles and formation of nucleocapsids all take place in the nucleus of the infected cell. Thereafter, DNA-containing capsids acquire a primary envelope when they enter the perinuclear space by budding into the inner nuclear membrane, followed by a subsequent de-envelopment process through the outer nuclear membrane [12]. Once in the cytoplasm, the nucleocapsids acquire their inner tegument [13]. Finally, virion assembly concludes through a secondary envelopment process by budding into trans-Golgi network (TGN)-derived vesicles coated with viral glycoproteins and more tegument proteins [14]. During this process, virions acquire the outer tegument and the envelope. Although this model of envelopment/de-envelopment/re-envelopment is widely accepted [15,16], many aspects of the process remain to be unravelled, specifically those concerning the molecular tools that HSV-1 uses to exploit the cellular trafficking machinery.

Small GTPase Rab27 [17-19] subfamily consists –in vertebrates– of two isoforms, Rab27a and Rab27b, which display a high homology. Both isoforms, although differing in cell type specificity, have been implicated in regulated exocytosis and might play a key role in certain events of membrane trafficking. Rab27a and Rab27b are functionally redundant but display differential expression in tissues: while Rab27a is mainly expressed in a broad range of secretory cells [20], melanocytes, endocrine cells and cytotoxic T lymphocytes (CTLs), Rab27b is expressed in platelets, endocrine cells, spleen and brain, being absent in melanocytes and CTLs [21]. Hence, Rab27a and Rab27b show different expression patterns, especially in brain, although both proteins are expressed in pituitary cells [22]. A recent work showed that downregulation of Rab27a blocked lysosomal exocytosis in Schwann cells and reduced the remyelination of regenerated sciatic nerve, suggesting an important role for Rab27a in remyelination within the peripheral nervous system [23]. In addition, a role for Rab27 isoforms in exosome secretion has also been demonstrated [24]. Rab27a was the first example of a Rab protein implicated in a human genetic disease: Griscelli syndrome type 2 (GS2), a rare autosomal recessive disorder caused by mutations in the Rab27a gene [25]. Clinical features of this syndrome include partial albinism and immune disorder. The ashen mouse is the corresponding murine model [26]. In accordance with the location of secretory granules, Rab27a is polarized towards the apical domain of epithelial cells [20].

Rab27a regulates secretion of lysosome-related organelles (LROs), a heterogeneous group of organelles which share features with multivesicular bodies (MVBs)/lysosomes. Nevertheless, although LROs share various features with late endosomes/lysosomes, they differ in function, morphology, and composition. These organelles include, among others, melanosomes in melanocytes, lytic granules in CTLs, dense granules in platelets, azurophilic granules in neutrophils and eosinophils and Weibel-Palade bodies (WPB) in endothelial cells [27,28]. Although all these cellular compartments share several characteristics, LROs and classic secretory granules differ in the source of their membrane and lumenal contents: most of LROs content derives from the endosomal system, whereas secretory granules derive directly from the TGN. However, it is now accepted that LROs comprise a very heterogeneous group of organelles that seem to have diverse origins [29].

Several Rab GTPases have been involved in the morphogenesis of herpesviruses. In particular, recent works have revealed the role for Rab1a/b, Rab3a and Rab43 in HSV-1 envelopment [30,31]. Other Rab proteins, such as Rab6 and Rab27a, have also been involved in HCMV –a member of the *betaherpesvirinae* subfamily– assembly [31-33]. Given the similarities in the assembly processes amongst several members of the *Herpesviridae*[10], we investigated the role of Rab27a in HSV-1 morphogenesis. We show that this small GTPase colocalizes in the TGN with the viral glycoproteins gH and gD, together with a pUL46-green fluorescent protein (GFP)-tagged HSV-1 (GHSV-UL46). Moreover, Rab27a depletion decreases the infection rate. Taken together, these data point to a significant role for Rab27a in the infection of oligodendrocytic cells with HSV-1.

## Results

### Expression of Rab27a in HOG cells

Several reports have previously shown Rab27a expression on many different cell types. However, to date, no study addressed Rab27a expression in oligodendrocytic cultures. For this purpose, the expression of this GTPase in the human HOG oligodendroglial model was investigated using RTqPCR, immunoblot analysis and confocal immunofluorescence microscopy.

**Figure 1:**
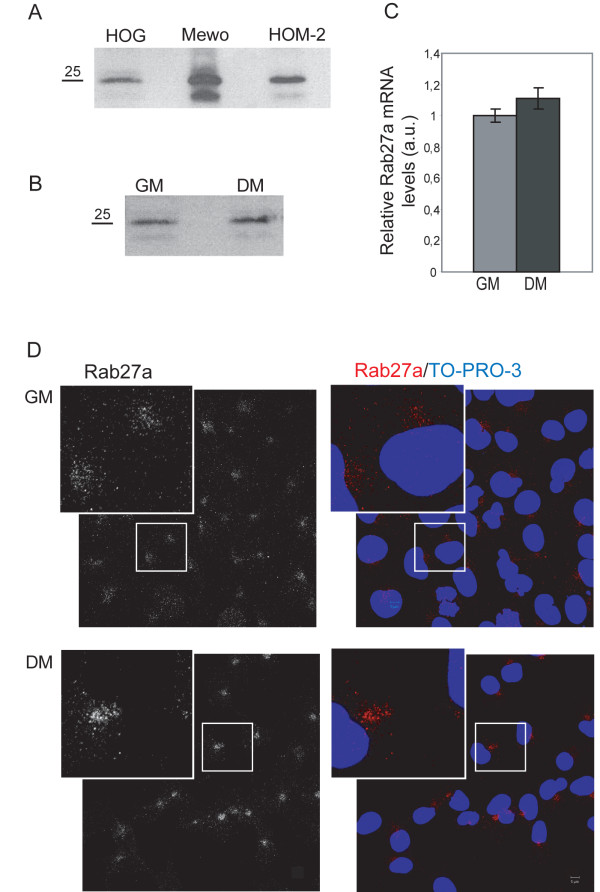
**Expression of Rab27a in HOG cell line.A**. HOG cells cultured in GM were subjected to SDS–PAGE under non-reducing conditions and analyzed by immunoblotting with anti-Rab27a polyclonal antibody. Compared to positive controls, Mewo and HOM-2 cell lines, HOG cells show significant levels of Rab27a expression. **B**. RTqPCR quantification of relative Rab27a mRNA expression levels in HOG cells cultured in GM or DM. **C**. Immunoblot analysis of Rab27a expression in HOG cells cultured in GM or DM. HOG cells were subjected to SDS–PAGE under non-reducing conditions and analyzed by immunoblotting with anti-Rab27a polyclonal antibody Immunoblot assays showed a moderate increase of Rab27a in DM cultures. **D**. HOG cells cultured in GM or DM were fixed and processed for confocal immunofluorescence analysis with anti-Rab27a polyclonal antibody, detected using an Alexa Fluor 555 secondary antibody. The squares correspond to enlarged regions showing pericentrosomal localization of Rab27a, more scattered in the case of GM cultures. Images correspond to the projection of the planes obtained by confocal microscopy. (DIC: Differential Interference Contrast). All data are representative of, at least, 3 independent experiments. (a.u., arbitrary units).

Immunoblot assays showed the expression of Rab27a in HOG cells. The Epstein Barr virus-transformed, human lymphoblastoid HOM-2 cells and the human melanoma MeWo cell line, which are known to express high levels of Rab27a [33], were used as positive controls. When compared with these two cell lines, HOG cells displayed a significant level of expression (Figure 1A). To further determine whether Rab27a expression was modified following cell differentiation, we first investigated the expression of Rab27a mRNA by RT-qPCR in cells cultured either in growth (GM) or differentiation medium (DM). In previous works, we have established the differentiation stage of HOG cell line under different conditions, showing that culturing cells for 24 hours in DM is sufficient to induce an increment in PLP expression and an enrichment of this protein in myelin-like sheets [34,35] Immunoblot assays showed a moderate increase of Rab27a in DM cultures (Figure 1B). Quantitative RT-PCR confirmed an approximate 10% increment of Rab27a expression in HOG cells cultured under differentiation conditions in comparison to GM cultured cells (Figure 1C).

To perform microscopy analysis, HOG cells cultured in DM were fixed and processed for confocal immunofluorescence analysis with an anti-Rab27a polyclonal antibody. An increase in Rab27a in differentiated compared to undifferentiated cells was also found. Rab27a was mostly detected in a region probably corresponding to the pericentrosomal area, although it was also detected in scattered cytoplasmic small vesicles (Figure 1D). More Rab27a-positive scattered vesicles were found in the cytoplasm of cells cultured in GM, although their location was also mainly pericentrosomal. Despite this observation, the pattern of of Rab27a distribution in cells cultured in DM was quite similar to that observed in cells cultured in GM. For this reason, we decided to show the results obtained only in differentiated cells, essentially analogous to the ones obtained with GM cultures.

### Subcellular localization of Rab27a

**Figure 2:**
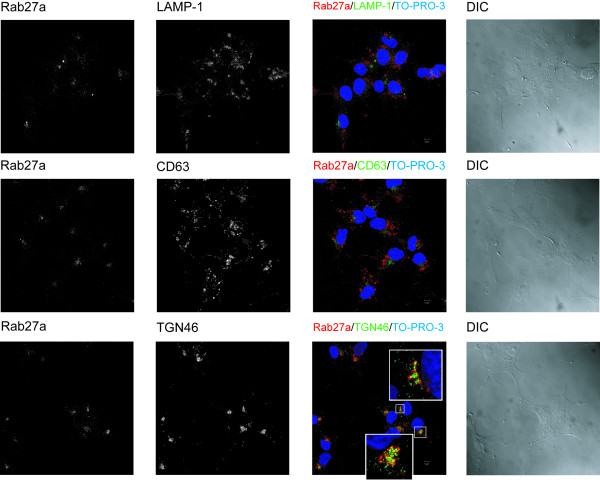
**Subcellular localization of Rab27a in HOG cells. A**. HOG cells cultured in DM were fixed and processed for confocal double-label indirect immunofluorescence analysis with anti-Rab27a polyclonal antibody and antibodies against LAMP-1, CD63 and TGN-46. Primary antibodies were detected using Alexa Fluor 555 and 488 secondary antibodies. Images correspond to the projection of the planes obtained by confocal microscopy. Colocalization (yellow spots) was detected between Rab27a and TGN-46. The squares show enlarged images corresponding to a confocal slice of 0.8 μm. (DIC: Differential Interference Contrast).

To study the subcellular localization of Rab27a in HOG cells, we performed further immunofluorescence analysis. To this aim, HOG cells cultured in DM were fixed and processed for confocal double-labeled indirect immunofluorescence analysis with primary antibodies. First of all, we tested lysosomal markers LAMP-1 and CD63, to assess the plausible colocalization of these proteins with Rab27a. However, in our hands, no colocalization was observed (Figure 2). Other markers, such as CD9 and TGN46, were tested as well. Among all of them, TGN46 seemed to be the only one displaying colocalization with Rab27a (Figure 2) (Manders coefficients: M1 = 0,89 M2 = 0,61).

### Expression and localization of Rab27a in HSV-1 -infected cells

As a first approximation to assess the feasible relationship between Rab27a and HSV-1, HOG cells cultured in DM were infected at a m.o.i of 1 with two GFP-tagged HSV-1, GHSV-UL46 and K26GFP. Subsequently, after infection, mRNA levels and location were determined by RTqPCR and confocal immunofluorescence microscopy analysis, respectively. Immunofluorescence microscopy analyses were carried out within 18 h p.i. RTqPCR analysis did not show significant changes in Rab27a expression within 8 h p.i. (data not shown).

**Figure 3:**
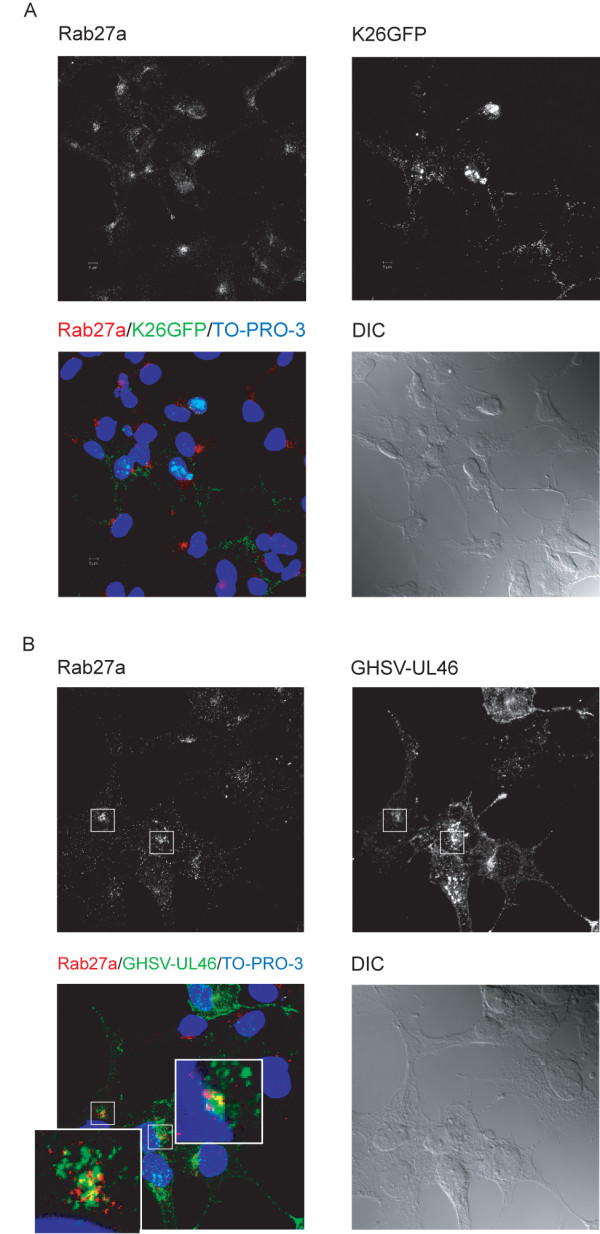
**Expression and localization of Rab27a in HSV-1-infected cells.** Triple-label indirect confocal immunofluorescence analysis of HOG cells infected with K26GFP (**A**) or GHSV-UL46 (**B**) with an anti-Rab27a polyclonal antibody, detected with an Alexa Fluor 555 secondary antibody. The squares show enlarged images corresponding to a confocal slice of 0.8 μm showing partial colocalization of GHSV-UL46 with Rab27a (yellow spots). (DIC: Differential Interference Contrast).

Comparative analysis between GHSV-UL46 and K26GFP infection showed that, unlike capsid-tagged K26GFP virus (Figure 3A), tegument-tagged GHSV-UL46 displayed partial colocalization with Rab27a (Figure 3B) (Manders coefficients: M1 = 0,72 M2 = 0,45). Absence of colocalization with capsids could be explained by the rapid transport of capsids at the TGN. Other studies have also shown that the relatively short life cycle of HSV-1 makes it difficult to analyze the vectorial movement of this virus during its rapid egress [36].

**Figure 4:**
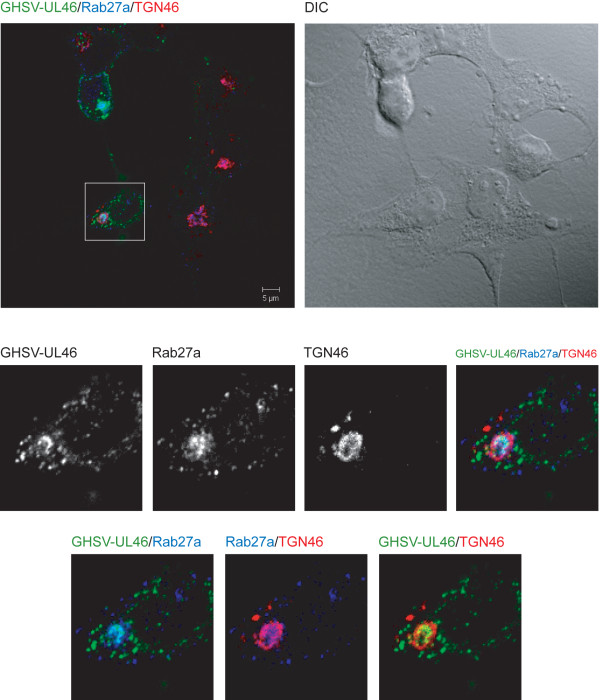
**Colocalization between GHSV-UL46 and Rab27a in the TGN.** HOG cells cultured in DM and infected at a m.o.i. of 1 with GHSV-UL46 were fixed and processed for confocal triple-label indirect immunofluorescence analysis with anti-Rab27a and anti-TGN-46 polyclonal antibodies. Low panels, corresponding to confocal slices of 0.8 μm, are enlargements of the square shown in upper panel, which corresponds to the projection of the planes obtained by confocal microscopy. Images show colocalization between Rab27a and GHSV-UL46 in the TGN. Colocalization between Rab27a and GHSV-UL46 appears cyan; between Rab27a and TGN, magenta; between GHSV-UL46 and the TGN, yellow; colocalization between Rab27a, GHSV-UL46 and TGN appears white. (DIC: Differential Interference Contrast).

In this regard, it is widely accepted that HSV-1 acquires tegument and envelope through a process of secondary envelopment by budding into TGN-derived vesicles coated with viral glycoproteins and tegument proteins. Since we found a significant colocalization between Rab27a and TGN, we carried out confocal triple-labeled indirect immunofluorescence analysis with anti-Rab27a and TGN46 antibodies, and GHSV-UL46 virus. Figure 4 shows partial colocalization between GHSV-UL46, Rab27a and TGN-46 (Manders coefficients of colocalization GHSV-UL46/TGN-46: M1 = 0,79, M2 = 0,70; GHSV-UL46/Rab27a : M1 = 0,7 M2 = 0,51; colocalization TGN/Rab27a : M1 = 0,77 M2 = 0,56).

**Figure 5:**
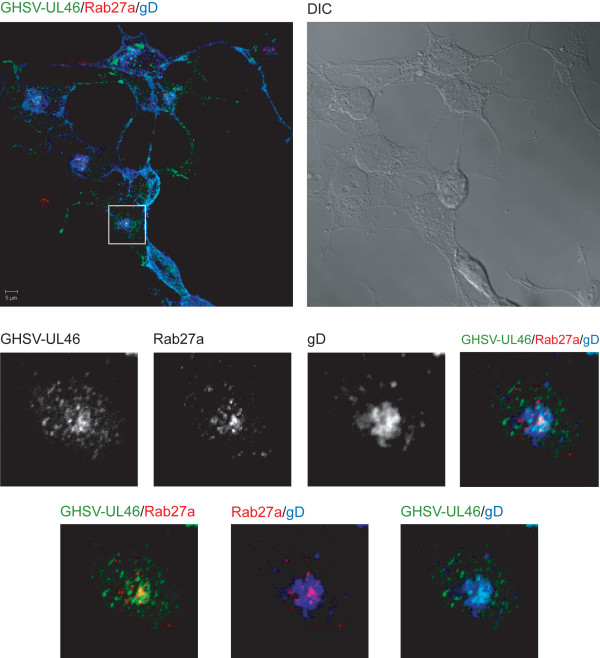
**Colocalization between Rab27a and gD.** HOG cells cultured in DM and infected at a m.o.i. of 1 with GHSV-UL46 were fixed and processed for confocal triple-label indirect immunofluorescence analysis with polyclonal anti-Rab27a and anti-gD LP2 antibodies. Low panels, corresponding to confocal slices of 0.8 μm, are enlargements of the squares shown in upper panels, which correspond to the projection of the planes obtained by confocal microscopy. Images show colocalization between Rab27a and gD. Colocalization between Rab27a and GHSV-UL46 appears yellow; between Rab27a and gD, magenta; between GHSV-UL46 and gD, cyan; colocalization between Rab27a, GHSV-UL46 and gD appears white. (DIC: Differential Interference Contrast).

**Figure 6:**
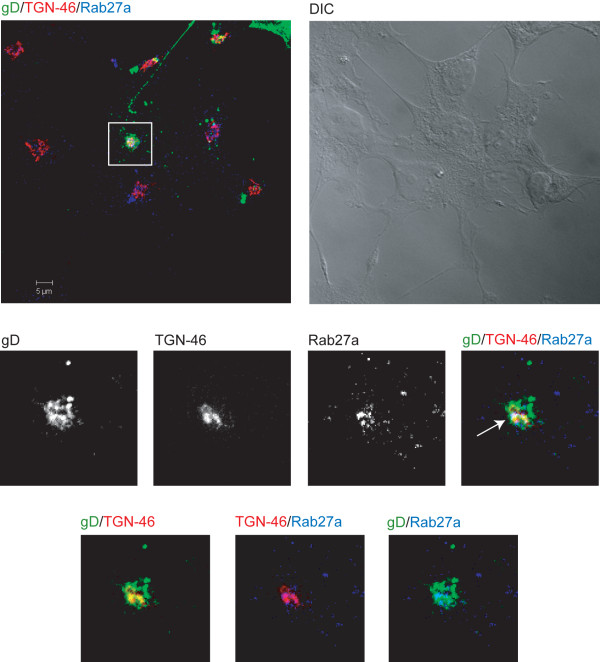
**Colocalization between Rab27a and viral glycoproteins in the TGN.** HOG cells cultured in DM and infected at a m.o.i. of 1 with wild-type HSV-1 were fixed and processed for confocal triple-label indirect immunofluorescence analysis with polyclonal anti-Rab27a and anti-TGN-46 antibodies. Low panels, corresponding to confocal slices of 0.8 μm, are enlargements of the squares shown in upper panels, which correspond to the projection of the planes obtained by confocal microscopy. Colocalization between gD and the TGN appears yellow; between Rab27a and the TGN, magenta; between Rab27a and gD, cyan. Arrow points to colocalization of Rab27a with gD in the TGN. (DIC: Differential Interference Contrast).

It has been shown that HSV-1 glycoproteins accumulate in the TGN and in TGN-derived vesicles [10]. Since we suspected a feasible role for Rab27a in viral morphogenesis, the next step was to assess whether Rab27a colocalized with viral glycoproteins. To this end, we performed confocal triple-labeled indirect immunofluorescence analysis with anti-Rab27a, anti-gH LP11 [37] and anti-gD LP2 [38] antibodies. As expected, both gH (data not shown) and gD colocalized with Rab27a (Figure 5) (Manders coefficients Rab27a/gD: M1 = 0,78 M2 = 0,7). Finally, triple-labeled indirect immunofluorescence analysis with antibodies anti-Rab27a, anti-gD LP2 and anti-TGN46 demonstrated that colocalization of this viral glycoprotein with Rab27a took place in the TGN (Figure 6) (Manders coefficients of colocalization gD/TGN-46: M1 = 0,7, M2 = 0,6; TGN/Rab27a : M1 = 0,7 M2 = 0,66; colocalization gD/Rab27a : M1 = 0,73 M2 = 0,59).

### Effect of Rab27a depletion in HSV-1 infection

**Figure 7:**
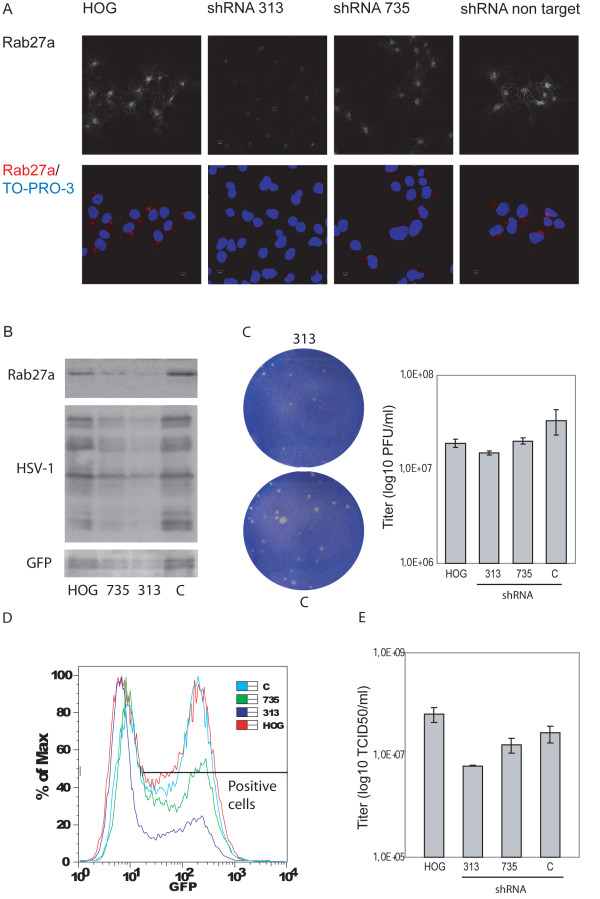
**Effect of Rab27a depletion on HSV-1 infection.** HOG cells mock-transfected or transfected with Rab27a-silencing shRNA-313 or shRNA-735, and shRNA non target control, were fixed and processed for confocal immunofluorescence analysis with polyclonal anti-Rab27a antibody. As images show, shRNA-313 induced an efficient knockdown of Rab27a while shRNA-735 produced a weaker effect (**A**). Equal number of cells were subjected to SDS–PAGE and analyzed by immunoblotting with anti-HSV-1 and anti-GFP antibodies. In Rab27a-depleted cells, a significant decrease in viral-associated GFP signal can be observed (**B**). Plaque assay shows a drastic reduction in plaque size and a decrease in the viral production determined by the number of plaque forming units (p.f.u.) per ml in silenced shRNA-313 cells compared to control cells (**C**). Silenced cells and controls infected at a m.o.i. of 1 with K26GFP were processed for flow cytometry, analyzing fluorescence of GFP (**D**). Percentage (%) of max designates the number of cells relative to the maximum fraction. For each fluorescence intensity within positive cells, the percentage of silenced cells corresponding to shRNA-313 and 735 is considerably lower than controls. Data are representative of 3 independent experiments. **E**. Rab27a-depleted cells and controls were infected at a m.o.i. of 0.5 with HSV-1. 18 h p.i., viral titers were determined by TCID_50_. Virus yield was significantly reduced in shRNA-313 silenced cells.

Further analysis of the role of Rab27a during HSV-1 infection, was carried out by shRNA knockdown. To generate stably silenced cell lines, HOG cultures were transfected with two plasmids expressing Rab27a shRNAs. One of them, named shRNA-313, induced an efficient knockdown of Rab27a while, in comparison, a second one, shRNA-735, elicited a weaker effect (Figure 7A and 7B).

Once our cellular model was established, susceptibility to HSV-1 infection was assessed by plaque assay and immunoblot analysis. In addition, viral entry was also investigated using a recombinant HSV-1 (gL86) which expresses β-galactosidase upon entry into cells. In Rab27a-silenced cells, an important decrease in viral-associated GFP signal was observed 18 h p.i. (Figure 7B). Plaque assay showed a drastic reduction in plaque size of silenced shRNA-313 cells compared to control cells (Figure 7C). Moreover, the number of plaques also decreased, suggesting that Rab27a depletion could be affecting the viral egress. Moreover, cells were infected at a m.o.i. of 1 with K26GFP and then, processed for fluorescence activated cell sorter (FACS) analysis. The number of GFP-expressing cells and their mean fluorescence were measured 24 hour after infection. As shown in Figure 7D, a significant decrease in these parameters was confirmed in Rab27a-silenced cells compared with non-target control shRNA-expressing and non-transfected cells. Histogram data have been expressed as percentage of maximum (% of max), in which Y axis corresponds to the number of cells for each fluorescence intensity of the X axis, relative to the peak fraction of cells. To assess whether Rab27a is involved in the viral cycle, we measured viral yield of infected cells. Viral titer of Rab27a-silenced infected cells also showed, within 24 h p.i., a significant decrease compared with non-target control shRNA-expressing and non-transfected cells (Figure 7E). This effect is not due to a differential entry capacity of virions into the cell, since kinetics of viral entry showed no difference among silenced and control cultures (data not shown). Altogether, these results suggest that Rab27a might be required not only in viral egress, but also in viral production.

## Discussion

Many details on the molecular mechanism utilized by HSV-1 to exploit the cellular trafficking machinery during morphogenesis are uncertain. In particular, several aspects regarding the process of the secondary envelopment and viral egress need further enlightenment. Final steps of viral assembly take place through secondary envelopment by budding into TGN-derived vesicles coated with viral glycoproteins and tegument proteins [10,11,36,39-41]. Herein, we suggest the involvement of the Rab-GTPase Rab27a in this process.

Various Rab GTPases have been involved in HSV-1 –as well as in other herpesviruses– envelopment [30-32]. In fact, Rab27a is required for assembly of HCMV [33]. Given the similarities among members of the herpesvirus family [10], we decided to analyze whether Rab27a plays any influential role in HSV-1 infection of oligodendrocytic cells.

First of all, our results showed a significant level of expression of Rab27a in HOG cells, compared to HOM-2 and MeWo cell lines, which were used as positive controls. Although we found a slight increase of Rab27a expression after culturing cells under differentiation conditions, results obtained in both systems (DM versus GM) did not differ substantially. Therefore, we decided to present only the results corresponding to differentiated cells, that is, cells cultured with DM.

To study the subcellular localization of Rab27a in our oligodendrocytic system, we performed confocal immunofluorescence microscopy analysis. For this purpose, and taken into account previous studies, we considered the analysis of lysosomal markers LAMP-1 and CD63, to check whether colocalization of these markers with Rab27a actually occurred. However, and contrary to previous findings [24,42-46], no colocalization could be observed. Interestingly, further experiments showed colocalization between Rab27a and TGN46.

Thus, in HOG oligodendroglial cells, Rab27a expression was mostly detected in a region surrounding what seems to be the pericentrosomal area displaying a positive signal for the TGN marker, TGN-46. To depict thoroughly the identity and features of the Rab27a-positive structure found in our model, further studies will have to be undertaken. However, given its lysosomal features, it was expectable to find a certain degree of colocalization between Rab27a and the late endosomal/lysosomal proteins LAMP1 and CD63, as it has been described in other systems.

Several previous findings may explain the absence of LAMP-1 and CD63 in Rab27a-positive structures and the colocalization of Rab27a with TGN-46. LROs comprise a heterogeneous group of organelles that share various features with late endosomes/lysosomes, but differ in function, morphology, and composition. The existence of a high variety of apparently related organelles, suggests that not all LROs share a common biogenetic pathway. Thus, LROs comprise a very heterogeneous group of organelles that seem to have diverse origins: for example, whereas melanosomes originate from early endosomes, WPBs emerge from the TGN. [29]. In addition, although the majority of LROs share certain characteristics, many of them display completely different features as well. Maturation stage of the cells must also be considered, since the recruitment of Rab27a is a dynamic process that depends on the maturation and polarization stage of the cell [45,47]. In this sense, for instance, when von Willebrand factor (VWF) is heterologously expressed in some cultured cell lines, such as HEK-293, it causes the formation of structures similar to WPBs that can recruit endogenous Rab27a. In HEK-293 cells, endogenous Rab27 was observed in a compact pericentriolar region probably corresponding to the microtubule organizing centre. This endogenous Rab27 did not show colocalization with LAMP1 suggesting that there was little or no enrichment of Rab27 on late endosomes/lysosomes. Nevertheless, in VWF expressing HEK-293 cells, significant enrichment of endogenous Rab27 was found on the VWF-containing WPB-like organelles that had formed. Thus, Rab27a was recruited specifically to the VWF-containing organelles and not to the lysosome in a maturation-dependent process that was independent of the cell type [47]. Therefore, the recruitment of Rab27a is a complex process driven by elements such as the maturation stage and the cargo molecules in which protein markers follow a dynamic pattern of expression and reorganization depending on those factors.

Once the study model was established, we investigated the relationship between Rab27a and HSV-1 infection. For this goal, HOG cells were infected with GHSV-UL46 and K26GFP. GHSV-UL46 is a tegument tagged HSV-1 [48], whereas K26GFP was obtained fusing GFP to a HSV-1 capsid protein [49]. After finding a high degree of colocalization between Rab27a and TGN, we proceeded to assess whether HSV-1 colocalized with Rab27a in that compartment. We found that Rab27a colocalized with tegument-tagged GHSV-UL46 in the TGN, whereas only a very low level of colocalization with capsid-tagged K26GFP was ascertained. This fact might be explained by the fast transit of capsids through the TGN during its rapid egress.

HSV-1 acquires tegument and envelope through a process of secondary envelopment by budding into TGN-derived vesicles coated with viral glycoproteins and tegument proteins. Consequently, we investigated whether viral glycoproteins were associated with Rab27a, finding that this small GTPase colocalized with viral glycoproteins gH and gD, and with GHSV-UL46. On the other hand, viral titer of Rab27a-silenced infected cells showed a significant decrease compared with non-target control shRNA-expressing and non-transfected cells, supporting the idea of an involvement of Rab27a in HSV-1 cycle. Finally, functional studies showed that Rab27a depletion produced a significant decrease on the infection rate. Analysis of the number of GFP-expressing cells 24 hours after infection with K26GFP virus, showed a significant decrease of these parameters in Rab27a-silenced cells compared to non-target control shRNA-expressing and non-transfected cells. Taken together, these results suggest a possible role for Rab27a in HSV-1 infection of oligodendrocytic cells. Also, the reduction of the size and number of viral plaques in silenced cells, points to an effect of Rab27a in the process of viral egress. Therefore, Rab27a might be involved in viral secretion. Since, colocalization between viral glycoproteins and Rab27a takes place in the TGN or in TGN-derived vesicles, and given that Rab27a depletion also induced a reduction in the viral production, we suggest that Rab27a might be required in both processes, viral morphogenesis and egress.

Finally, our results show that Rab27a depletion reduced both the viral production and viral egress, effect that is not due to a differential entry capacity of virus. Therefore, the reduction in the cell-associated infectious viruses under Rab27a shRNA silencing, and the colocalization between viral glycoproteins and Rab27a in the TGN, suggest that Rab27a might be relevant for virus morphogenesis, maybe for secondary envelopment.

## Conclusions

Our work suggests a role for the small Rab GTPase Rab27a during HSV-1 infection. First of all, herein we show for the first time the expression of this small GTPase in oligodendroglial cells. In spite of the fact that several Rab GTPases have been involved in the morphogenesis of herpesviruses, no data about the role of Rab27a in HSV-1 infection has been reported to date.

Microscopy studies demonstrated partial colocalization of Rab27a with viral glycoproteins in the TGN. Moreover, viral titer of Rab27a-silenced infected cells showed a significant decrease compared with control cells. In addition, functional analysis confirmed a significant decrease of GFP-expressing cells 24 hour after infection of Rab27a-silenced cells with a GFP-tagged HSV-1. Reduction of the size and number of viral plaques in Rab27a-depleted infected cells, points to an effect of this protein in the process of viral egress. On the other hand, colocalization between viral glycoproteins and Rab27a takes place in the TGN or in TGN-derived vesicles, and given that Rab27a depletion also induced a reduction in the viral production, we suggest that Rab27a might be required in viral morphogenesis and egress.

## Methods

### Antibodies and reagents

Horseradish peroxidase-conjugated secondary anti-IgG antibodies were from Millipore (Billerica, MA, USA). Anti-LAMP1 mouse monoclonal antibody H4A3 was from DSHB (Developmental Studies Hybridoma Bank, University of Iowa, USA). Anti-Rab27a rabbit polyclonal antibody [50] was kindly provided by Dr P. van der Sluijs, (University Medical Center Utrecht, The Netherlands). Sheep anti-TGN-46 polyclonal antibody was from Serotec. Anti-GFP rabbit polyclonal serum A6455, Alexa 488-, Alexa 647- and Alexa 594-conjugated secondary antibodies were obtained from Molecular Probes (Eugene, OR, USA). Low-glucose DMEM, fetal bovine serum (FBS), o-nitrophenyl-β-D-galactopyranoside (ONPG), carboxymethylcellulose sodium salt (CMC) medium-viscosity and protease inhibitor cocktail were purchased from Sigma Chemical Co. (St. Louis, MO, USA). Mowiol was from Calbiochem (Merck Chemicals, Germany). Jet-PEI was from Polyplus-transfection (Illkirch, France).

### Cell lines and virus

The human HOG cell line, established from a surgically removed human oligodendroglioma [30] was kindly provided by Dr. A. T. Campagnoni (University of California, UCLA, USA). Cells were cultured on Petri dishes in GM containing low-glucose DMEM supplemented with 10% fetal bovine serum (FBS), penicillin (50 U/mL) and streptomycin (50 μg/mL) at 37°C in an atmosphere of 5% CO_2_. To induce differentiation, cells were cultured in serum-free DM containing low-glucose DMEM supplemented with additives [35].

The Epstein Barr virus-transformed, human lymphoblastoid cell line HOM-2 was generously provided by Dr. M. Izquierdo (Instituto de Investigaciones Biomédicas “Alberto Sols”, Madrid, Spain). MeWo cells were a kind gift of Dr. L. Montoliu (CNB, Madrid, Spain).

GHSV-UL46, purchased from ATCC (American Type Culture Collection), is a recombinant HSV-1 labeled by fusing GFP to a structural protein of its tegument (VP11/12), the product of the UL46 gene [48]. K26GFP was a kind gift of Dr. Desai (Johns Hopkins University, Baltimore, USA). It was obtained by fusing GFP to the HSV-1 capsid protein VP26 [49]. Viruses were propagated and titrated on Vero cells. HSV-1 (KOS) gL86, a b-galactosidase–expressing version of KOS strain [51], was propagated in 79VB4 cells, a Vero-derived cell line stably expressing gL. CHO-K1 and 79VB4 cells, and HSV-1 (KOS) gL86, were a kind gift of Dr. R. Longnecker (Northwestern University, Chicago, USA).

### Immunoblot analysis

Equal number of cells were subjected to SDS-PAGE in 12% acrylamide gels and transferred to Immobilon-P membranes (Millipore). To detect Rab27a, electrophoresis was performed under non-reducing conditions. After blocking with 5% nonfat dry milk 0.05% Tween 20 in PBS, blots were incubated for 1 hr at room temperature with primary antibodies. After several washes with 0.05% Tween 20 in PBS, blots were incubated for 1 hr with secondary antibodies coupled to horseradish peroxidase, washed extensively, and developed using an enhanced chemiluminescence Western blotting kit (ECL, Amersham, Little Chalfont, UK).

### RNA-interference mediated silencing

HOG cells were transfected with plasmids expressing shRNAs previously described [33]. Transfection protocol was performed as described [34]. Briefly, twenty-four hours prior transfection of the HOG cell line, one million cells were plated in 100 mm tissue culture dishes with GM. Cells were transfected with 3 μg of DNA, using the JetPEI reagent according to the manufacturer’s instructions. Cells were incubated with DNA for 24 h in GM and, 48 h after transfection, selection of stable HOG cell transfectants was carried out by treatment with GM containing 2 mg/ml puromycin, and Rab27a silencing was analyzed by immunoblot. Plasmids encoding non-target control (SHC002) and Rab27a shRNAs TRCN0000005294 (313) and TRCN0000005295 (735) were from Sigma (MissionH TRC-Hs shRNA libraries, Sigma Aldrich).

### Viral infections

For viral infection assays, 1.2 × 10^6^ HOG cells growing in 60-mm tissue culture dishes were mock infected or infected with the corresponding virus. During viral adsorption, cells were maintained in DMEM with antibiotics in the absence of FCS. Subsequently, cultures were rinsed and cultured in DM. Viral titer was quantified by an endpoint dilution assay determining the 50% tissue culture infective dose (TCID_50_) in Vero cells, considering the final dilution that shows cytopathic effect and using the Reed and Muench method.

For plaque assay, confluent monolayers of cells plated in 6-well tissue culture dishes were infected with serial dilutions of HSV-1. After viral adsorption, cells were washed and overlayed with CMC. The CMC solution was prepared in distilled water at 2% (w/v) and stirred at room temperature for one hour. CMC overlay (1% final concentration) was prepared by mixing equal volumes of CMC 2% and DM double-strength. Two millilitres of CMC overlay were added to each well. Plates were incubated at 37°C in a humidified 5% CO_2_ incubator for 48 hours. After that, CMC overlay was aspirated and cells were washed with PBS. Plaques were visualized by staining with crystal violet.

### Entry assay

To determine HSV-1 entry, confluent monolayers of HOG cells plated in 96-well tissue culture dishes were infected with serial dilutions of recombinant HSV-1 (KOS) gL86, which expresses β-galactosidase upon entry into cells. After 6 h p.i., β-galactosidase assays were performed using a soluble substrate ONPG assay. The enzymatic activity was measured at 410 nm using a Benchmark microplate reader (Bio Rad). HSV-1 resistant CHO-K1 cells were used as control.

### Real-time quantitative RT-PCR assay

Total RNA from triplicate samples of HOG cells cultured in 60-mm dishes under growth or differentiation conditions was extracted using RNeasy Qiagene Mini kit (Qiagen, Valencia, CA, USA). RNA integrity was evaluated on Agilent 2100 Bioanalyzer (Agilent Technologies, Santa Clara, CA). Then, RNA was quantified in a Nanodrop ND-1000 spectrophotometer (Thermo Fisher Scientific). All the samples showed 260/280 ratio values around 2, which correspond to pure RNA. Yield range was between 405 and 639 ng/μl. RNA Integrity Number (RIN) values were between 9.3 and 9.8, corresponding to RNA samples with high integrity. Genomic DNA contamination was assessed by amplification of representative samples without retrotranscriptase (RT).

RT reactions were performed using the High Capacity RNA-to-cDNA Master Mix with No-RT Control (Applied Biosystems PN 4390712) following manufacturer’s instructions. Briefly, 1 μg of total RNA from each sample was combined with 4 μl of master mix (including all necessary reagents among which a mixture of random primers and oligo-dT for priming). RT- controls were obtained by using the No-RT master mix included in the master mix pack. The reaction volume was completed up to 20 μl with DNAse/RNAse free distilled water (Gibco PN 10977). Thermal conditions consisted of the following steps: 5’ × 25°C, 30’ × 42°C and 5’ × 85°C. RT- amplifications of the representative samples were either negative or delayed more than 5 cycles compared to the corresponding RT + reactions. Intron-spanning assays were designed using Probe Finder software (Roche Applied Science). Primer sequences were as follows: 5’-AGGCCAGAGAATCCACCTG-3’ (forward), and 5’-GCATCTCTGAAGAACGCTGTC-3’ (reverse). Manufacturer of oligonucleotides was Sigma Aldrich. Oligo design, RT-qPCR and data analysis was performed by the Genomics Core Facility at Centro de Biología Molecular Severo Ochoa (CSIC-UAM).

In order to know the most suitable genes for the normalization, the stability of four candidates –β-Actin, GAPDH, 18S and UBQ– were assayed using the NormFinder algorithm. Given its exceptionally high stability, 18S was chosen as the most appropriate. On the contrary, β-Actin showed the highest instability through the process of differentiation.

qPCR reactions were performed in triplicates in a final volume of 10 μl with a cDNA amount equivalent to 10 ng of total RNA, 500 nM of each primer and 5 μl of SsoFast EvaGreen SuperMix (Bio-Rad, CN 172-5204), according to the manufacturer’s instructions. For all the genes we carried out an initial denaturation of 30’’ × 95°C followed by 40 two-step cycles (5’’ × 95°C + 5’’ × 60°C). We also included a melting curve from 60°C to 95°C (0.5°C/seg) at the end of the program to verify the specificity of the PCR. Fluorescence was acquired during both the 60°C and melting steps. Reactions were set up robotically, with an Eppendorf pipetting robot (epMotion 5075). qPCR instrument was a CFX384 Real Time System C1000 Thermal Cycler (Bio-Rad). No Template Control (NTC) amplifications were always either negative or delayed more than 5 cycles with respect to the experimental samples.

In order to estimate the individual efficiency of each primer pair and to validate a quantitative range for each assay we performed a qPCR over a six-point ¼ dilution curve made from a “pool” cDNA sample (cDNA input range equivalent to 50-0.05 ng total RNA). The quantification cycles (Cqs) of the experimental samples were within the ranges validated by the dilution curves.

### Flow cytometry analysis

To perform FACS analysis, HOG cells were dissociated by incubation for 1 minute in 0.05% trypsin/0.1% EDTA (Invitrogen) at room temperature and washed and fixed in 4% paraformaldehyde for 15 minutes. Then, cells were rinsed and resuspended in PBS. Cells were analyzed using a FACSCalibur Flow Cytometer (BD Biosciences).

### Immunofluorescence microscopy

Cells grown on glass coverslips were fixed in 4% paraformaldehyde for 20 min, rinsed with PBS and treated with 20 mM glycine for 5 min to quench aldehyde groups. Cells were then permeabilized with 0.2% Triton X-100, rinsed and incubated for 30 min with 3% bovine serum albumin in PBS with 10% human serum, to block the HSV-1-induced IgG Fc receptors. For double and triple-labeled immunofluorescence analysis, cells were incubated for 1 hr at room temperature with the appropriate primary antibodies, rinsed several times and incubated at room temperature for 30 min with the relevant fluorescent secondary antibodies. Antibodies were incubated in the presence of 10% human serum. Controls to assess labeling specificity included incubations with control primary antibodies or omission of the primary antibodies. After thorough washing, coverslips were mounted in Mowiol. Images were obtained using an LSM510 META system (Carl Zeiss) coupled to an inverted Axiovert 200 microscope. Quantification of colocalization, was performed using M1 and M2 Manders coefficients [52]. We calculated Manders overlap coefficients selecting regions of interest corresponding to the areas where the colocalization seemed to be high, that is, areas in yellow, magenta and cyan. Processing of confocal images and colocalization analysis was made by FIJI-ImageJ software.

## Competing interests

The authors declare that they have no competing interests.

## Authors’ contributions

RB-M performed the experiments and wrote the manuscript. AJC carried out the viral infections and titrations. ET and AA participated in the experimental design and helped to edit the manuscript. JAL-G and AF-R conceived and designed the study, and participated in experimental design. JAL-G coordinated the study and edited the manuscript. All authors read and approved the final manuscript.
